# Hemoglobin quantification in red blood cells via dry mass mapping based on UV absorption

**DOI:** 10.1117/1.JBO.26.8.086501

**Published:** 2021-08-10

**Authors:** Nischita Kaza, Ashkan Ojaghi, Francisco E. Robles

**Affiliations:** a Georgia Institute of Technology, School of Electrical and Computer Engineering, Atlanta, Georgia, United States; b Georgia Institute of Technology and Emory University, Wallace H. Coulter Department of Biomedical Engineering, Atlanta, Georgia, United States

**Keywords:** deep-UV microscopy, mass mapping, quantitative phase imaging, red blood cells, hemoglobin, hematology analysis

## Abstract

**Significance:** The morphological properties and hemoglobin (Hb) content of red blood cells (RBCs) are essential biomarkers to diagnose or monitor various types of hematological disorders. Label-free mass mapping approaches enable accurate Hb quantification from individual cells, serving as promising alternatives to conventional hematology analyzers. Deep ultraviolet (UV) microscopy is one such technique that allows high-resolution, molecular imaging, and absorption-based mass mapping.

**Aim:** To compare UV absorption-based mass mapping at four UV wavelengths and understand variations across wavelengths and any assumptions necessary for accurate Hb quantification.

**Approach:** Whole blood smears are imaged with a multispectral UV microscopy system, and the RBCs’ dry masses are computed. This approach is compared to quantitative phase imaging-based mass mapping using data from an interferometric UV imaging system.

**Results:** Consistent Hb mass and mean corpuscular Hb values are obtained at all wavelengths, with the precision of the single-cell mass measurements being nearly identical at 220, 260, and 280 nm but slightly lower at 300 nm.

**Conclusions:** A full hematological analysis (including white blood cell identification and characterization, and Hb quantification) may be achieved using a single UV illumination wavelength, thereby improving the speed and cost-effectiveness.

## 1 Introduction

The morphological and molecular properties of blood cells are important indicators of health and are diagnostic markers for several diseases.^1^^–^^7^ The morphology, size, and hemoglobin (Hb) content of red blood cells (RBCs) serve as essential biomarkers to diagnose or monitor various types of anemias and other diseases, including malaria and thalassemia.^5^^–^^7^ RBCs are enucleated, terminally differentiated cells that are composed of 95% to 98% Hb by dry mass (mass of all the constituents of a cell in the absence of water).^8^^–^^10^ Thus, the dry mass of RBCs is of clinical significance, and hematology analyzers routinely used to obtain a complete blood count (CBC) from peripheral blood samples measure indices such as the dry mass of Hb in the form of the mean corpuscular Hb (MCH), and the mean corpuscular Hb concentration.^7^ Along with mean values, single-cell measurements are invaluable to completely characterize a sample’s distribution.^11^^,^^12^ In many clinical hematology analyzers, Hb concentration is obtained from the optical scattering of spherical cells.^13^ Owing to the characteristic biconcave shape of RBCs, they have to be treated with a sphering agent, which lengthens the procedure and leaves cells in a nonphysiological state.^14^^,^^15^ In addition, in some pathological conditions, such as sickle cell anemia, the RBCs are difficult to sphere.^16^

Label-free optical techniques to quantify the dry mass and other physical properties of individual cells^10^^,^^15^^,^^17^^–^^22^—in their natural state without requiring additional pretreatment or sample preparation—offer an excellent alternative and have been successfully applied to quantify Hb in RBCs.^5^^,^^6^^,^^15^^,^^21^^–^^24^ For example, quantitative phase imaging (QPI)-based methods^5^^,^^6^^,^^17^^–^^19^^,^^21^^,^^23^ measure the phase accumulated in biological samples and translate this metric into dry mass density by leveraging the linear relationship between refractive index and mass concentration.^17^ QPI methods gained popularity due to their accuracy, sensitivity, and speed, but they lack molecular specificity and cannot clearly distinguish between the different organelles or cellular compartments having different chemical compositions.^25^

Deep-ultraviolet (UV) microscopy has emerged as a high-resolution, label-free imaging technique capable of providing quantitative molecular information due to the distinctive absorptive properties of endogenous biomolecules in this region of the spectrum.^20^^,^^24^^,^^26^^–^^28^ Deep-UV microscopy images have been used to successfully generate accurate nucleic acid and protein mass maps of live cells owing to their intrinsic molecular contrast, without the need for laborious fixing and staining procedures, ^10^^,^^20^^,^^22^^,^^26^ we recently demonstrated that deep-UV microscopy images acquired at 300 nm could accurately measure Hb mass.^24^ Moreover, a five-part white blood cell (WBC) differential count was achieved by analyzing absorption and morphological features at 260 nm, and a colorization scheme was devised, which could recapitulate the gold-standard Giemsa stained appearance of blood cells using images acquired at 260, 280, and 300 nm.

UV absorption-based mass mapping has multiple advantages over QPI-based methods. First, UV mass maps have higher spatial resolution than typical QPI-based measurements due to the UV light’s shorter wavelength. In addition, while a number of QPI systems rely on an interferometric setup to measure the phase, intensity-based UV imaging systems can have simpler instrumentation (they do not require beam splitters, gratings, etc.) and have better light throughput (i.e., they do not have to split the light source to obtain a reference field). Most importantly, UV absorption-based mass mapping is able to distinguish and quantify the different chemical constituents of a cell rather than grouping them together, as in QPI-based mass maps.

The accuracy of UV-absorption-based Hb mass mapping depends on the wavelength, and oxygenation-state dependent absorption properties of this molecule. In this letter, we present a comparison of RBC mass mapping, and thereby Hb quantification, at four UV wavelengths: 220, 260, 280, and 300 nm using transmission microscopy images from our multispectral UV imaging system.^24^ Note that 220 and 280 nm correspond to regions of high protein absorption, 260 nm corresponds to the absorption peak of nucleic acids, whereas absorption at 300 nm is only significant for Hb. Our previous study^24^ leveraged this insight and used 260 nm for WBC identification and 300 nm for Hb analysis. However, imaging protocols could be greatly simplified if only a single wavelength was necessary to achieve both WBC and RBC (and thus Hb) characterization.

In addition to comparing data acquired at four different UV wavelengths, we also compare our results to measurements obtained from QPI-based mass mapping using interferometric data from our UV hyperspectral interferometric (UHI) microscopy system.^26^ We demonstrate consistent hemoglobin mass quantification at all wavelengths and obtain MCH values lying within the normal reference range (29.5±2.5  pg).^7^ The single-cell mass measurements from UV absorption-based mass maps have the highest precision (measured in terms of the standard deviation) at 220 nm, closely followed by 260 and 280 nm and slightly lower at 300 nm. These results indicate that a full hematological analysis may be achieved using a single UV illumination wavelength.

## 2 Materials and Methods

The multispectral UV microscopy system depicted in Fig. S1 in Supplemental Material uses a laser-driven plasma source (EQ-99X LDLS, Energetiq Technology) that is relayed to the sample through an off-axis parabolic mirror (Newport Corporation) and a short-pass dichroic mirror (Thorlabs). A filter wheel fitted with UV band-pass filters (∼10  nm FWHM, Chroma Technology) enables multispectral imaging at four center wavelengths: 220, 260, 280, and 300 nm. A 40×, 0.5 NA UV-objective (LMU-40X, Thorlabs) is used for imaging, which provides an average spatial resolution of ∼280  nm. The images at each wavelength are captured using a UV-sensitive CCD camera (pco.UV, PCO AG). We note that commercially available LEDs at 260, 280, and 300 nm can replace the broadband source and filter wheel, reducing the cost, complexity, and footprint of our system.

We compare the results from UV absorption-based mass mapping with QPI-based mass mapping using data acquired from our UHI microscopy system^26^ shown in Fig. S2 in Supplemental Material. This system uses a 4f interferometric configuration in a Mach–Zehnder geometry with the same light source, to enable coherent detection of optical fields.^26^ The same 40× UV objective is used for imaging but data are collected with an imaging spectrometer (IsoPlane-160, Princeton Instruments) equipped with a back-illuminated sCMOS camera (Kuro 1200, Princeton Instruments). One spatial dimension of the sample is captured along the rows of the camera, whereas its spectrum is recorded along the columns. The sample is then line-scanned using a high-precision motorized stage (MLS2031, Thorlabs) to obtain a hyperspectral data-cube. The interferometric configuration and the use of beam splitters limit the light-throughput and do not allow imaging at wavelengths of interest below 240 nm where the source power is low (e.g., at 220 nm, where all proteins have significant absorption). Thus, this system enables broadband hyperspectral imaging with quantitative phase, absorption, and dispersion information from 240 to 450 nm. While the UHI system has access to more detailed spectral information compared with the simpler brightfield, multispectral UV microscope system, the UHI system is bulkier, and can be far more challenging to align (owing to the low spatial and temporal coherence of the source). Therefore, each method has its unique strengths with UHI being more appropriate for research, whereas the brightfield, multispectral UV system is more apt for clinical/translational applications.

The optical density (OD(x,y)) of a UV-transmission image at a specific x-y location depends on the measured intensity ($I_{m}(x,y)$) at that location, and the intensity of a blank field ($I_{0}(x,y)$) according to the Beer-Lambert law.^10^^,^^20^ The blank field is obtained by capturing an image without the sample, keeping all other imaging conditions (e.g., camera’s integration time) unchanged (this compensates for any system or detector spectral dependence). The OD is thus defined as

$$\mathrm{OD}(x,y)=-\log_{10}(\frac{I_{m(x,y)}}{I_{0}(x,y)}).$$ (1)

Here, we take the OD to be equal to the absorbance.^20^ The typical OD values for RBCs are in the approximate range 0.69 to 0.81 at 220 nm, 0.1 to 0.22 at 260 nm, 0.12 to 0.24 at 280 nm, and 0.03 to 0.15 at 300 nm. The absorption at 220, 260, and 280 nm is high from both Hb and other membrane proteins. Thus, an effective molar extinction coefficient for RBCs can be calculated using a weighted average of the extinction coefficients for Hb^29^ (95%) and an “average protein”^20^^,^^22^ (5%) (whose extinction coefficient is calculated using the procedure detailed in Ref. 20; deviations in this value will have minimal impact on the overall mass). Although the Hb content may vary between 95% and 98% of the dry mass, this small variation will have a proportionally small effect (≤3%) on the Hb mass estimate. As reported in Ref. 22, the effective ε values used for RBCs are: $\varepsilon_{220}=606,000\;L{\mathrm{mol}}^{-1}\;{\mathrm{cm}}^{-1}$, $\varepsilon_{260}=112,360\;L{\mathrm{mol}}^{-1}\;{\mathrm{cm}}^{-1}$, and $\varepsilon_{280}=115,634\;L{\mathrm{mol}}^{-1}\;{\mathrm{cm}}^{-1}$. A detailed explanation of the calculation of these effective values is provided in the Supplemental Material. At 300 nm, we assume that Hb is the only significant absorber and that contributions from the membrane proteins are negligible, and hence $\varepsilon_{300}=\varepsilon_{{\mathrm{Hb}}_{300}}=65,972\;L{\mathrm{mol}}^{-1}\;{\mathrm{cm}}^{-1}$. Hence, the dry mass per unit area (σ) of the RBC is given as

$$\sigma_{{\mathrm{RBC}}_{\lambda }}(x,y)=\frac{\mathrm{OD}(x,y)}{\varepsilon_{\lambda }}M,$$ (2)

where M is the molar mass of the constituents. Based on the previous assumptions, at 220, 260, and 280 nm, the dry mass of Hb is 95% of the total dry mass obtained from absorption-based measurements, i.e., $\sigma_{{\mathrm{Hb}}_{\lambda }}=0.95\sigma_{{\mathrm{RBC}}_{\lambda }}$. Similarly, M in Eq. (2) is 63,911  g/mol a weighted average of the molar masses of hemoglobin (64,500  g/mol) and the “average” protein (52,728  g/mol). At 300 nm, since all the absorption is assumed to be from Hb, we have $\sigma_{{\mathrm{Hb}}_{\lambda }}=\sigma_{{\mathrm{RBC}}_{\lambda }}$ and $M=M_{Hb}=64,500\;g/\mathrm{mol}$.

A similar set of assumptions needs to be made for mass mapping with QPI. The phase accumulated as light travels through a sample depends on the wavenumber, the difference between the refractive indices of the sample and surroundings, and the sample thickness. The refractive index of a cell at a given wavelength ($n_{\text{cell}}(\lambda )$) is related to its concentration (c) as $n_{\text{cell}}(\lambda )=n_{m}(\lambda )+c\beta_{\lambda }$, where $\beta_{\lambda }$ is the refractive index increment at a specific wavelength and $n_{m}$ is the refractive index of the surrounding medium, which is considered to be very similar to the refractive index of the cytoplasm, for cells suspended in media.^5^ Therefore, the dry mass per unit area is related to the phase via

$$\sigma_{\lambda }(x,y)=\frac{\lambda }{2\pi \beta_{\lambda }}\phi_{\lambda }(x,y),$$ (3)

where ϕ is the measured phase.^19^^,^^30^ Here, we follow established QPI-based methods for assessing RBCs mass maps, which do not consider contributions from proteins.^6^ Accordingly, we use refractive index increments for Hb reported in Ref. 26, averaged over a 10-nm bandwidth. Hence, we have $\beta_{260}=0.2561\;\mathrm{mL}/g$, $\beta_{280}=0.2304\;\mathrm{mL}/g$, and $\beta_{300}=0.2183\;\mathrm{mL}/g$.^26^ The total mass of the cell (from both methods) is computed from the mass density as

$$\text{mass}=\underset{x}{\sum }\underset{y}{\sum }\sigma_{\lambda }(x,y).ps,$$ (4)

where ps is the pixel size and the x and y coordinates cover the entire cell area.

## 3 Results and Discussion

**Fig. 1:**
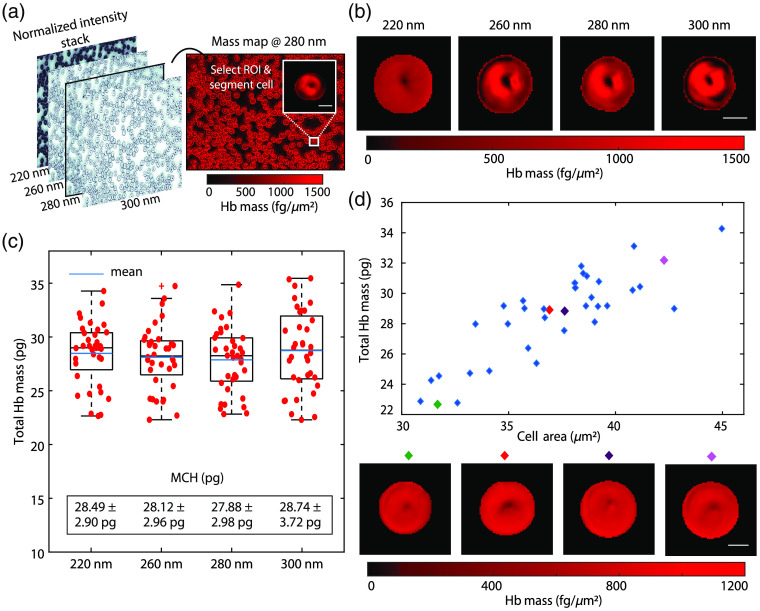
(a) UV absorption-based mass mapping workflow. A mass map is generated corresponding to each normalized and coregistered intensity in the stack, followed by the selection of a rectangular ROI and cell segmentation. (b) Mass maps at 220, 260, 280, and 300 nm from a typical RBC. (c) Box plot showing distributions of 36 RBCs at 220, 260, 280, and 300 nm. MCH values are listed on the plot. (d) Distribution of total masses with cell area at 220 nm. Example cells are shown below the plot. (Scale bars: 5  μm).

For UV absorption-based mass mapping, whole blood is collected from a healthy donor according to protocols approved by the Institutional Review Board of Georgia Institute of Technology and Emory University. A blood smear is prepared and imaged, after air drying for 5 min, at the four different wavelengths using the multispectral UV microscopy setup. The intensity images at each wavelength, normalized by the blank reference, are coregistered using a simple intensity-based registration algorithm implemented in MATLAB (Mathworks). The mass map is calculated at each wavelength using Eq. (2). Unphysical values of mass per area, such as negative values, are thresholded. The intensity stack at the four wavelengths and the associated mass map at 280 nm are shown in Fig. 1(a), where the high absorption at 220 nm compared to the other wavelengths can be clearly seen (cells appear very dark in the grayscale intensity maps).

To analyze individual RBCs, we identify rectangular regions of interest (ROIs) containing representative cells (N=36) that have minimal to no overlap with surrounding cells. Then, the RBCs are further segmented using Otsu’s thresholding method, followed by morphological operations including erosion, dilation, opening, and closing using a disk element to refine the mask and avoid edge (diffraction) artifacts arising from some degree of spatial coherence of the light source^31^ and wavelength-dependent aberrations. This segmentation is repeated for the same set of cells at all four wavelengths and yields unique masks at each wavelength, which accounts for slight variations in the images (e.g., from wavelength-dependent aberrations). A fully automated analysis pipeline that uses more sophisticated algorithms would greatly improve our throughput and will form part of our future work.

The mass maps of a typical healthy RBC at the four wavelengths are shown in Fig. 1(b). While they are generally similar, small variations in morphological features are possibly due to a combination of aberrations, lensing effects,^32^^,^^33^ and diffraction artifacts. At 220 nm, strong absorption from both membrane proteins and Hb results in a homogeneous mass map with a high contrast-to-noise ratio (CNR). The mass maps at 260 and 280 nm are very similar, but mass maps extracted from 280 nm illumination are slightly more uniform because of the relatively higher absorption from proteins and Hb at 280 nm compared to 260 nm. At 300 nm, Hb absorption is the lowest of the four wavelengths under consideration, and protein absorption is negligible, resulting in a noisier mass map (i.e., a worse CNR).

The boxplot in Fig. 1(c) shows the distributions of the total cell masses at the four wavelengths obtained using Eqs. (2) and (4) (the pixel size is $0.0216\;\mu m.^{2}$). At 220, 260, and 280 nm, the total masses have comparable ranges, mean, and median values. The MCH (total dry mass averaged across all the cells) is the highest at 300 nm (28.74 pg) and is the lowest at 280 nm (27.88 pg), with no statistically significant differences between the four wavelengths (using a two-tailed t-test). The standard deviation of the Hb dry mass is lowest at 220 nm (2.90 pg), very similar at 260 and 280 nm, and highest at 300 nm (3.72 pg), yielding a coefficient of variation (CV) ∼10%. The CV of the Hb mass distribution may be assumed to be equivalent to the red cell distribution width (based on correlation analyses in Ref. 23), which is typically 11% to 16%.^34^ Since we consider a relatively small number of cells, we may be unable to capture the variation across the entire patient RBC population, and the difference in the standard deviations of the Hb masses at different wavelengths may be due to broadening of the Hb mass distribution due to measurement errors. The total cell mass is plotted against the cell area in Fig. 1(d), and we observe that both the areas and masses are within the range of values for healthy RBCs.^22^^,^^24^ Mass maps from certain representative cells of different sizes are shown below the plot.

The UV absorption-based mass mapping method is directly dependent on the accuracy of the extinction coefficient values applied in the calculations. For Hb absorption, 300 nm is an isosbestic point (absorption is approximately equal for all forms of Hb), whereas the molar extinction coefficient at other wavelengths varies with oxygenation. Furthermore, while there is significant absorption from proteins at 220, 260, and 280 nm, the absorption at 300 nm is dominated by Hb alone, leading to the expectation that Hb-quantification at 300 nm would be most precise.^24^ The absorption coefficient at 220 nm has not been accurately measured or widely reported, and here we adopted the value determined empirically in Ref. 22. Despite these shortcomings, we find that the Hb masses at 220 nm have high CNR and have the lowest standard deviation (i.e., highest precision). Similar results are also obtained at 260 and 280 nm. On the contrary, because of the lower CNR, mass mapping at 300 nm performs the worst, defying previous expectations. Theoretically, it is possible to combine the measurements at different wavelengths to obtain robust estimates of the mass of Hb and non-Hb proteins, but this is hindered by imperfect pixelwise registration and wavelength-dependent aberrations. Moreover, the focus of this work is on Hb quantification using single-wavelength measurements (here our baseline ground truth is based on established QPI methods). UV microscopy images acquired at 260 nm have inherent nuclear contrast due to the absorption peak of nucleic acids and can capture all the features necessary for a five-part WBC differential.^24^ The results presented in this work demonstrate that the Hb quantification of RBCs at 260 nm is precise and reliable. These results are significant because they indicate that label-free hematology analysis can be achieved using only a single wavelength, which can lead to the development of a faster, more compact, and cost-effective point-of-care hematology analyzer.

**Fig. 2:**
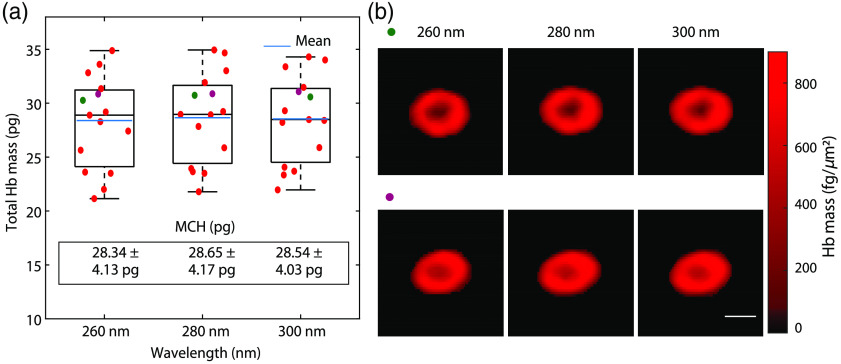
(a) QPI-based mass mapping. (a) Box plot showing distributions of 15 RBCs at 260, 280, and 300 nm. MCH values are listed on the plot. (b) Example mass maps of typical healthy RBCs (Scale bar: 5  μm).

Next, we compare the results obtained with UV absorption-based mass mapping to QPI-based mass mapping, which is a more well-established approach for dry mass mapping.^5^^,^^6^^,^^17^^–^^19^ To this end, isolated RBCs suspended in PBS are imaged with our UHI setup at a center wavelength of 275 nm, which gives us access to a spectral range of ∼250 to 325 nm. The resultant hyperspectral data-cube is processed to produce hyperspectral phase images.^26^ The phase images of 15 RBCs are averaged over a 10-nm bandwidth with center wavelengths 260, 280, and 300 nm to mimic the effect of the bandpass filters in our multispectral setup and ensure fair comparisons. Due to the limited throughput of the interferometric configuration, data at 220 nm are not available for comparison. The phase images at each wavelength are segmented using Otsu’s thresholding method, followed by morphological operations (described previously). This segmentation process provides the same treatment as with the UV absorption-based maps above. The total mass is obtained from the segmented mass map using Eq. (4) with a pixel size of $0.1\;\mu m^{2}$. The distributions of the total masses at each wavelength are shown by the boxplots in Fig. 2(a). The MCH is ∼28  pg (within the healthy reference range^7^), with statistically insignificant variations across wavelength. Similarly, the standard deviations of the single-cell Hb mass as measured across all wavelengths are ∼4  pg, with statistically insignificant variations across wavelength. Some example mass maps are shown in Fig. 2(b), which clearly highlight the typically biconcave morphology of healthy RBCs. The mass maps at the different wavelengths have nearly identical morphology, and differences in the masses at different wavelengths likely arise from noise fluctuations at each wavelength.

**Fig. 3:**
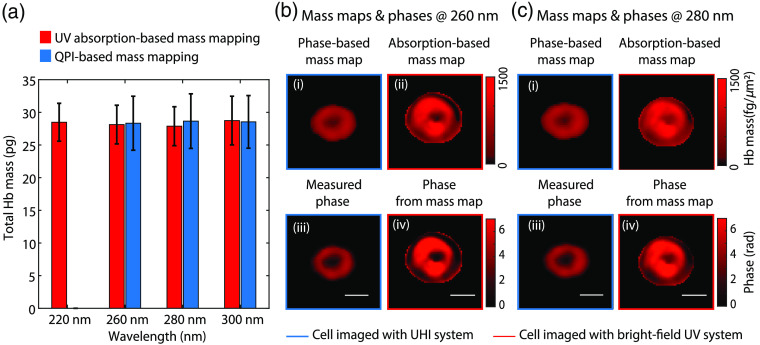
(a) Comparison of Hb mass from both mass mapping methods. (b) Phase maps obtained from mass maps of a cell imaged with the bright-field UV system compared with QPI-based mass maps and phases obtained using the UHI system of a similar cell at 260 nm. (c) Phase maps obtained from mass maps of a cell imaged with the brightfield UV system compared with QPI-based mass maps and phases obtained using the UHI system of a similar cell at 280 nm: (i) QPI-based mass map, (ii) UV absorption-based mass map, (iii) phase from UHI data, and (iv) phase from UV absorption-based mass map. (Scale bars: 5  μm).

The MCH values obtained from the two methods (referred to as UV and QPI henceforth) are compared in Fig. 3(a) and are in excellent agreement at 260, 280, and 300 nm (QPI-based data is not available at 200 nm). While no ground truth is available for the MCH (since a CBC is not performed for a blood sample from a healthy donor), we take the agreement between the MCH values obtained with both methods and the well-accepted healthy MCH reference range as an indication that our measurements are producing realistic results. The dry mass measurements from UV absorption-based mass mapping have a lower standard deviation (2.96 versus 4.13 pg at 260 nm) compared to the QPI-based method. This discrepancy may be a result of the biological variation (as different cells are imaged with both methods), and the smaller available sample size for the QPI-based method. Notwithstanding, the UV absorption-based mass maps have a higher spatial resolution owing to the noninterferometric, intensity-based imaging system configuration compared to the UHI system, which may contribute to an improved precision in Hb mass mapping. We note that this advantage will persist for our bright-field, intensity-based UV system versus more conventional QPI systems, which operate in the visible spectral range. While a high spatial resolution is not critical to estimate the total Hb mass of a cell, the improved spatial resolution of morphological images and mass density maps may be beneficial for disease diagnosis^5^^,^^6^ or aid in the study of RBC dynamics.^35^

Finally, given the linear relationship between optical phase and dry mass [as described by Eq. (3)], intensity-based UV mass maps may be translated to optical phase. To this end, we use the mass maps at a specific UV wavelengths, along with the β value at that those wavelengths (known *a priori*), to deduce optical phase information, following Eqs. (2) and (3). Figures 3(b) and 3(c) (ii and iv) show representative mass maps and phases (as derived from their initial UV mass estimate by effectively simulating the cells in PBS) at 260 and 280 nm for the same cell. Similarly, the mass maps obtained from phase measurements for a different cell imaged with UHI microscopy are shown for comparison in Figs. 3(b) and 3(c) (i and iii). The phase measurements reflect the expected biconcave shape of the cells at both wavelengths. We note that the images from the two systems have slightly different resolutions and magnification, giving rise to some of the observed differences. The phase values from the UV absorption-based mass maps do appear slightly higher, which could be largely due to biological variation. In addition, changes in the mass distribution of Hb in the smears as compared to the cells in media as well as inaccuracies in the optical constants used to calculate the phases could contribute to the differences in the observed phase values.

In conclusion, we have presented UV absorption-based mass mapping of individual RBCs and Hb quantification at four wavelengths: 220, 260, 280, and 300 nm. The estimated MCH values at the different wavelengths show no statistically significant differences, and the standard deviations of the mass measurements are nearly identical at 220, 260, and 280 but slightly higher at 300 nm. These results are surprising given that (1) 300 nm is an isosbestic point for Hb, which one would expect to remove potential variability in the extinction coefficient due to oxygenation levels, and (2) there are no other significant absorbers at this wavelength. Our data, however, indicate that the lower absorption at 300 nm leads to an overall lower CNR and hence a less precise measurement of Hb content. In addition, despite potential variability in oxygenation and contributions from other absorbers, RBC and Hb characterization at 220, 260, 280 nm provide more precise results. Further, in this work, we compared our results to QPI-based mass mapping, which is well accepted, and found excellent agreement on average between the two methods. Finally, we have demonstrated that our UV absorption-based mass maps can be translated into phase maps, from which other phase-related metrics can be computed. Therefore, deep-UV microscopy enables high-resolution, label-free morphological imaging, and accurate mass mapping and Hb quantification. Importantly, deep-UV microscopy images acquired at a single-wavelength (260 nm) can be used for characterizing RBCs and WBCs, thus enabling fast and cost-effective hematology analysis that can result in a simpler, point-of-care hematology analyzer.
