# Supplementary material for: Hemoglobin quantification in red blood cells via dry mass mapping based on UV absorption
Source: J Biomed Opt. 2021 Aug 10;26(8):086501. doi: 10.1117/1.JBO.26.8.086501 (PMC8353376; doi:10.1117/1.JBO.26.8.086501)
Supplement: Supplementary file 1 [file JBO_026_086501_SD001.pdf]

Supplementary Material for Hemoglobin quantification in red blood cells via dry mass mapping based on UV absorption

Nischita Kaza[a], Ashkan Ojaghi[b], Francisco E. Robles[b],*

a. School of Electrical and Computer Engineering, Georgia Institute of Technology, Atlanta, Georgia, USA

b. Wallace H. Coulter Department of Biomedical Engineering, Georgia Institute of Technology and Emory University, Atlanta, Georgia, USA.

*Correspondence should be addressed to F.E.R. (francisco.robles@bme.gatech.edu).

Effective molar extinction coefficient for RBCs

At 220, 260, and 280 nm, an effective molar extinction coefficient for RBCs can be calculated using a weighted average of the extinction coefficients for Hb [29] (95%) and an 'average protein' [20,22] (whose extinction coefficient is calculated using the procedure detailed in Ref. 20). The extinction coefficients of Hb used to compute the effective values are ε_{280} = 118,872 M^{-1} cm^{-1} i.e. (corresponding to deoxygenated Hb) and ε_{260nm} = 116,376 M^{-1} cm^{-1} (corresponding to oxygenated Hb). The molar extinction coefficients of oxyHb and deoxyHb are almost identical at 220, 260, and 300 nm; however, at 280nm there is an approximately 10% difference. While somewhat counterintuitive, these assumptions, are well-rooted in expected biophysical phenomena. Firstly, RBCs' oxygenation states at atmospheric pressure, outside of the body and without tight pressurization control, are not 100% oxygenated [36]. Additionally de/oxy-Hb rapidly evolve to metHb (the extinction coefficient of deoxyHb is intermediate to that of metHb and oxyHb at 280 nm [37]) outside of the body, including in-vitro with UV exposure [27]. These conditions are in line with the assumptions made Ref 22 (also validated here with our experiments and analyses) and provide a more consistent set of results compared to more intuitive assumptions of all RBCs being 100% oxygenated.

We note that dividing the extinction coefficients of Hb the 'average protein' by their masses and using a weighted average of these mass-specific extinction coefficient values to calculate the effective extinction coefficients causes a negligible change in the mass estimate since the masses of Hb and the protein are similar.

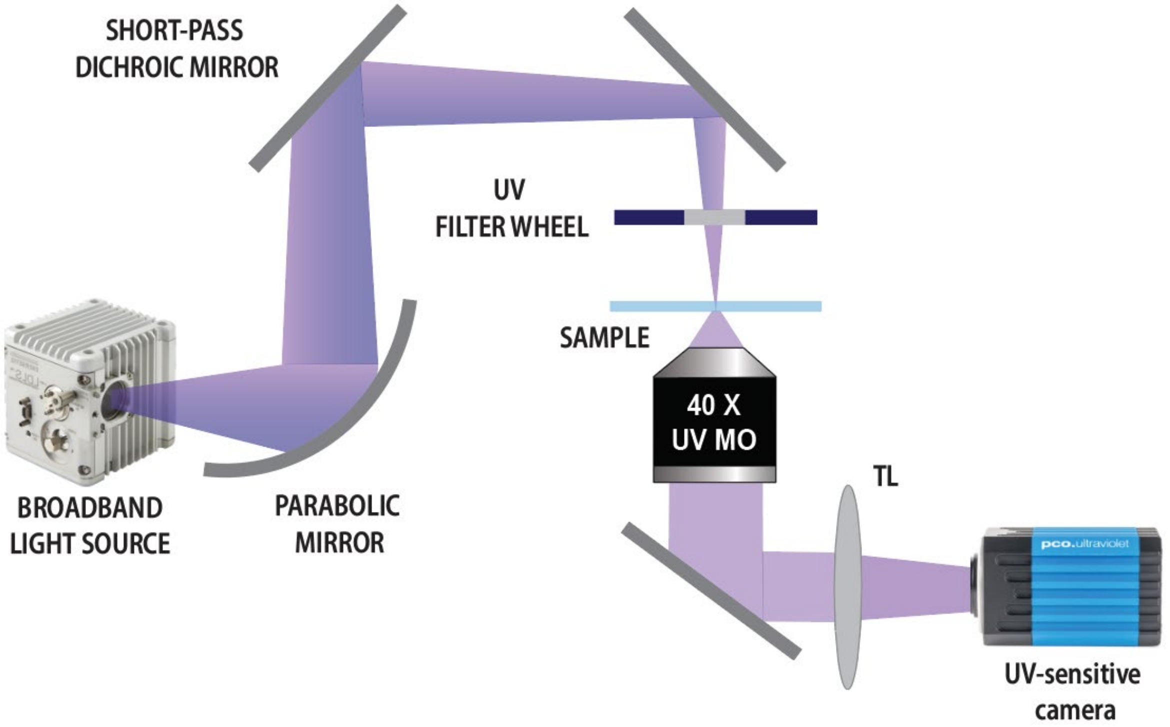

Fig. S1. Schematic of the multi-spectral deep-UV microscope consisting of a broadband plasma source, off-axis parabolic mirror and short-pass dichroic mirror to relay the light, and UV band-pass filters, a UV microscope objective, and UV-sensitive camera to enable multi-spectral imaging.

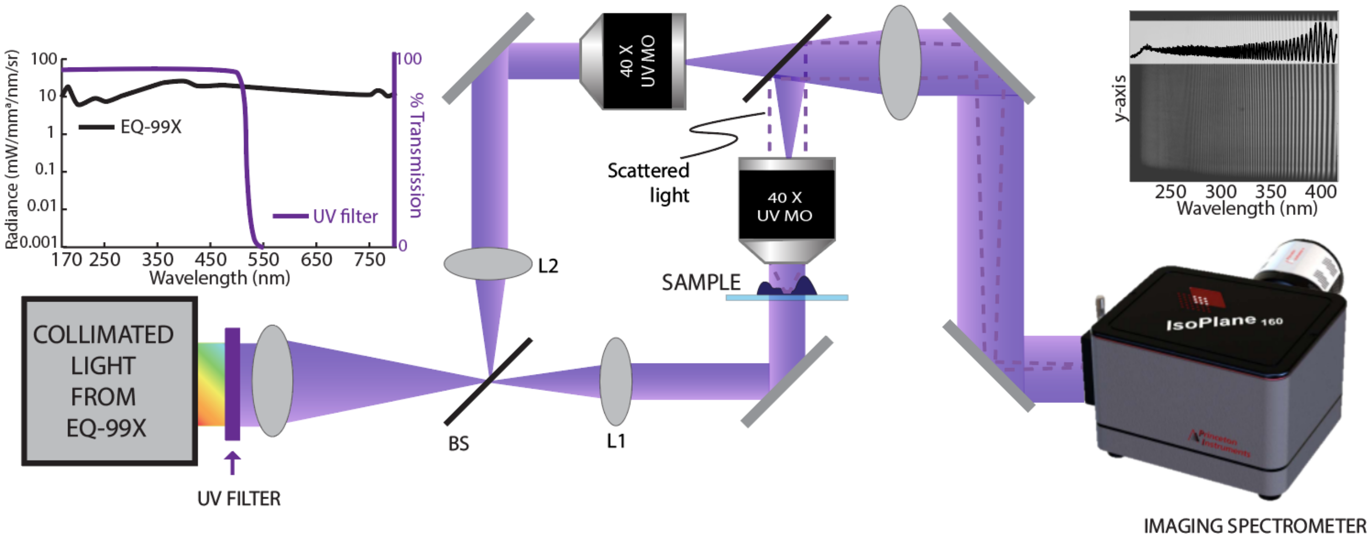

Fig. S2. Schematic of the UHI microscopy setup that used a collimated broadband spurce (radiance spectrum of the source output light is depicted in the left inset). The beam is split into reference and sample beams via the first beam splitter (BS) and collimated using L1 and L2 lenses. The imaging is performed using UV microscope objective (UV-MO) and the interferometric data (a sample image is shown in the right inset) is recorded by the imaging spectrometer.
